# Cryoablation combined with immunotherapy: new strategies and progress in the treatment of tumors

**DOI:** 10.3389/fonc.2026.1900725

**Published:** 2026-08-14

**Authors:** Yuxin Xiang, Lin Yu, Jia Li

**Affiliations:** 1 School of Clinical Medicine, Chengdu Medical College, Chengdu, China; 2 Department of Pulmonary and Critical Care Medicine, The First Affiliated Hospital of Chengdu Medical College, Chengdu, China; 3 Key Laboratory of Geriatric Respiratory Diseases of Sichuan Higher Education Institutes, Chengdu, China

**Keywords:** combination immunotherapy, cryoablation, immunotherapy, interventional therapy, tumors

## Abstract

In recent years, continuous advances in interventional therapies for pulmonary diseases have made minimally invasive treatment an important focus of this field because of its limited trauma, rapid recovery, and reliable efficacy. As a representative minimally invasive modality, cryoablation kills tumor cells mainly through cold-induced cellular injury, vascular injury, and immune modulation. Although existing studies have confirmed the immune-activating effects of cryoablation, cryoablation alone is limited by insufficient antitumor immune intensity and restricted control of distant tumors, making it difficult to maximize systemic antitumor efficacy. Recent evidence suggests that combining cryoablation with immunotherapy may amplify antitumor effects through complementary mechanisms. However, unlike previous reviews that have focused primarily on general mechanisms and preliminary efficacy, few have delved into the translational bottlenecks of cryoablation parameters, treatment sequencing, and lymph-node management. In addition to summarizing the immunological mechanisms, research progress, clinical applications, and current challenges, this review goes beyond mere description by synthesizing evidence on these three underappreciated aspects, proposing a practical sequencing framework, and outlining actionable strategies for lymph-node preservation versus ablation. We further appraise the evidence tumor by tumor and grade it by evidence tier, so that the strength of every recommendation is explicit and the tumor types for which no clinical data exist are named. Together these elements offer a clearer and more clinically instructive perspective for combination immunotherapy and provide a theoretical basis for optimizing treatment strategies for patients with tumors.

## 1 Introduction

Malignant tumors have become a major public health problem that seriously threatens human health. The incidence of early-onset cancer (diagnosed in people under 50 years old) is rising all over the world. Breast cancer, colorectal cancer and other common cancers are more aggressive in young people, which suggests that tumor prevention and control is facing new challenges. Although traditional surgery is the first-line treatment for early-stage solid tumors, it faces significant challenges in clinical practice: approximately 80% of patients with hepatocellular carcinoma are precluded from surgery due to multifocal lesions (1), insufficient hepatic functional reserve, or tumor adjacency to major vessels; the incidence of postoperative complications in pancreatic cancer can be as high as 57% (2); in non-small cell lung cancer, about 30% of patients are diagnosed at locally advanced stages, with a considerable proportion having contraindications to surgery (3); and patients with renal and prostate cancers often cannot tolerate radical resection due to advanced age or severe comorbidities (4, 5). These practical limitations make minimally invasive interventional therapy an important alternative treatment option for a broad spectrum of patients with solid tumors. For these patients, minimally invasive interventional therapy has become an important alternative treatment option. For example, in lung tumors, cryoablation has been regarded as a representative technology of minimally invasive treatment of lung, which directly destroys tumor tissue through the circulation process of low-temperature freezing-rewarming. Compared with traditional thermal ablation, cryoablation has the advantages of less pain, less damage to surrounding normal lung tissue (6, 7). After cryoablation, it was found that it can release tumor-associated antigens and damage-related molecular patterns (DAMPs) *in situ*, which has natural immune activation potential and provides new treatment possibilities for patients with unresectable NSCLC (8). However, in recent years, clinical practice and basic research have found that the application of cryoablation alone has obvious limitations: on the one hand, the endogenous anti-tumor immune response induced by cryoablation is often offset by the immunosuppression signal in the tumor microenvironment, resulting in a weak distal anti-tumor effect, which makes it difficult to remove circulating tumor cells and tiny metastases (9); on the other hand, the remodeling of the local immunosuppressive microenvironment after cryoablation may even promote the immune escape of residual tumor cells, leading to an increased risk of local recurrence or distant metastasis (9). This clinical bottleneck greatly limits the benefit of cryoablation in tumor treatment.

To overcome these limitations, the synergistic strategy of “cryoablation combined with immunotherapy” has become a research focus in the field of cancer in recent years. Tumor antigens released by cryoablation can function as an *in situ* vaccine and enhance the efficacy of immune checkpoint inhibitors and other immunotherapies. Immunotherapy, in turn, can relieve immunosuppression and expand tumor-specific T cells, further amplifying the systemic anti-tumor effect of cryoablation. Through such complementary mechanisms, the combination may achieve an anti-tumor effect greater than the sum of its parts (10). Basic studies have already confirmed clear synergy between the two approaches, and multiple early clinical studies have also suggested survival benefits. However, key clinical issues— including patient selection, treatment timing, and adverse-event management—remain controversial. Meanwhile, previous reviews have largely been confined to general mechanisms and preliminary efficacy, with limited exploration of the translational bottlenecks that truly affect clinical decision-making. Whereas most existing articles provide descriptive summaries of published trials, the present review distills evidence into three actionable clinical frameworks: (1) how cryoablation parameters (freezing rate, freeze–thaw cycles, and temperature) modulate the quality and magnitude of immune responses; (2) the optimal sequencing of combined therapy, with disease-state-specific recommendations; and (3) the management of tumor-draining lymph nodes, balancing local control against preservation of immune priming sites. These three aspects are directly relevant to the design of individualized treatment regimens and represent the primary added value of this review relative to the existing literature. On this basis, we briefly summarize the basic evidence, research progress, and current clinical applications of cryoablation combined with immunotherapy in tumors, point out existing challenges, and discuss future perspectives, aiming to provide a theoretical reference for optimizing treatment strategies.

Cryoablation has been investigated across a broad range of malignant tumors, and this review accordingly covers the current evidence in multiple cancer types. We place particular emphasis on non-small cell lung cancer (NSCLC) and hepatocellular carcinoma (HCC)—not to restrict the scope, but because they currently offer the most mature and robust evidence: (i) both have high incidence and large unresectable populations; (ii) cryoablation is technically most established in these settings; and (iii) the cryoablation–immunotherapy combination has shown the most consistent survival benefits here. Beyond these two, we further summarize the emerging evidence in melanoma, renal, breast, prostate, and pancreatic cancers, where data—though currently derived mainly from preclinical or early-phase clinical studies—are rapidly accumulating and support the broader applicability of this strategy across solid tumors. By presenting the evidence in a tiered manner across tumor types, this review aims to provide a clinically oriented reference framework that balances focused depth with comprehensive coverage.

## 2 Overview of cryoablation

### 2.1 Direct physical tumor destruction

Cryoablation achieves rapid cooling of target tissues via the Joule–Thomson effect. During the procedure, a percutaneously placed cryoablation probe delivers high-pressure liquefied gas (e.g., argon) precisely into the tumor. As the gas rapidly expands and transitions to a gaseous state, it absorbs heat and lowers the local temperature to approximately −140 °C, forming a visible ice ball that encompasses the target area. The ice ball boundary and coverage can be monitored in real time using computed tomography, magnetic resonance imaging, or ultrasound (11, 12).

The temperature within the ice ball exhibits a marked spatial gradient, giving rise to multiple synergistic physical destructive mechanisms. In the central lethal zone, extreme cooling promotes intracellular ice crystal formation, which directly and mechanically disrupts cell membranes and organelles. In the peripheral zone, extracellular ice formation elevates osmotic pressure, prompting water efflux from cells and subsequent dehydration; upon thawing, cells undergo osmotic swelling and rupture. Recrystallization during the rewarming phase further enlarges ice crystals and exacerbates membrane damage (6, 13, 14). Vascular injury constitutes another core mechanism: low temperatures damage microvascular endothelial structures, increase capillary permeability, and induce local edema and tissue swelling. Reperfusion injury after thawing can trigger platelet aggregation, microthrombus formation, and local ischemia, extending the zone of tissue necrosis beyond the immediate mechanical effects of ice crystals (14, 15). Collectively, these processes generate a spatially heterogeneous ablation zone—characterized by irreversible necrosis in the central region, a mixed zone of necrosis and programmed cell death in the intermediate area, and a sublethal margin where damaged but potentially recoverable cells may persist. This spatial architecture underscores that adequate tumor coverage and sufficient safety margins are essential for local control; conversely, incomplete ablation may not only leave viable tumor cells behind but also create a wound-healing microenvironment with immunosuppressive tendencies, thereby potentially compromising subsequent immune responses (6, 13).

Compared with thermal ablation modalities such as radiofrequency ablation, cryoablation causes less disruption to collagenous structures, theoretically offering advantages for tumors adjacent to major vessels or critical organs. It is also associated with less intraprocedural pain and enables real-time ice ball monitoring, attributes that confer unique clinical operational benefits.

### 2.2 Modulation of the tumor immune microenvironment

Beyond its direct physical tumoricidal effects, cryoablation exerts immunomodulatory actions through the release of tumor antigens and damage-associated molecular patterns (DAMPs) following cellular destruction. Studies have shown that cryoablation preserves the immunogenicity of tumor antigens and activates antigen-presenting cells via pattern recognition receptor signaling, thereby initiating T cell–mediated antitumor immune responses (16). However, the immunological effects elicited by cryoablation are bidirectional, and their magnitude and duration are influenced by multiple factors, including ablation parameters, tumor type, and host immune status. Moreover, the systemic immune activation induced by cryoablation alone is generally insufficient to effectively control distant metastases (9).

## 3 Overview of immunotherapy

Tumor immunotherapy kills tumor cells mainly by activating and strengthening the host immune system. Because it acts on the immune system itself, immunotherapy has a broad scope of application and can exert durable, systemic, and profound tumor-killing effects (17). Immunotherapy began to be explored clinically in lung cancer as early as the beginning of the twenty-first century. Since 2015, immune checkpoint inhibitors have been applied in lung cancer, including PD-1/PD-L1 inhibitors and CTLA-4 inhibitors, which have shown favorable therapeutic effects (18).

At present, in addition to immune checkpoint inhibitor monotherapy, an increasing number of immunotherapeutic agents are being used in combination for lung cancer treatment. Examples include studies of the safety and efficacy of bevacizumab combined with atezolizumab and platinum-based agents (19), the safety and efficacy of the novel targeted agent sacituzumab tirumotecan combined with immune checkpoint inhibitors (20), and studies comparing high-precision radiotherapy, such as stereotactic body radiotherapy (SBRT), with radiotherapy combined with the immunotherapeutic agent atezolizumab (21). These approaches aim to further improve survival benefits, optimize immunotherapy strategies, and provide more precise immunotherapy options for a broader population of patients with NSCLC.

## 4 Immunological mechanisms of cryoablation

### 4.1 Tumor antigen release

Cryoablation forms ice crystals within tumor cells through freeze–thaw cycles, disrupts cell membrane structures, causes necrosis, and releases large quantities of cellular components. Physical ice crystals mechanically destroy cells and directly disrupt the cell membrane. At the same time, they trigger two programmed cell death pathways. One is the mitochondrial apoptotic pathway, in which an imbalance in the BAX/BCL-2 protein ratio induces mitochondrial outer membrane permeabilization, promotes cytochrome c release, and activates the caspase-9/3 cascade. The other is the necroptotic pathway, in which the RIPK1/RIPK3 complex mediates phosphorylation of MLKL and ultimately leads to cell membrane rupture.

However, the term “immunogenic cell death” should not be used as a catch-all for every type of cryo-induced cell death. Necrosis, apoptosis, necroptosis, and pyroptosis differ markedly in terms of membrane integrity, inflammatory mediators, and antigen processing, and they elicit distinct immune outcomes (22, 23). Their relative contributions vary with ablation settings and tissue context. Uncontrolled necrosis can release DAMPs but may also produce immunosuppressive mediators; apoptosis is generally tolerogenic unless secondary necrosis intervenes; and while necroptosis and pyroptosis are lytic and potentially immunogenic, direct clinical evidence supporting their therapeutic relevance in human cryoablation remains scarce (22, 24). Hence, the immunological impact of cryoablation cannot be attributed to a single cell-death mechanism. In the central lethal zone, unregulated necrosis predominates, characterized by rapid cell membrane rupture and massive release of tumor antigens and DAMPs, which facilitates local inflammation and antigen uptake. However, extensive necrosis may also release high concentrations of IL-6, TGF-β, adenosine, oxidized lipids, and cellular debris, potentially impairing dendritic cell function or recruiting immunosuppressive myeloid cells. Thus, a larger necrotic area does not necessarily translate into stronger systemic immune activation. In the peripheral sublethal zone, apoptosis is the dominant form of cell death, with relatively intact cell membranes and encapsulated cellular contents within apoptotic bodies. Efficient clearance of apoptotic cells generally exerts anti-inflammatory or immunotolerant effects; only when clearance is delayed and secondary necrosis occurs may inflammatory signals be re-released.

Necroptosis is a regulated lytic cell death process mediated by the RIPK1/RIPK3 and MLKL axis. It can release DAMPs while retaining programmed signaling context, thereby supporting dendritic cell activation and cross-presentation. A cryo-nanocatalyst study enhanced necroptosis and achieved local PD-L1 inhibition in preclinical models, but this does not prove that routine clinical cryoablation typically induces therapeutically relevant necroptosis (22). Pyroptosis depends on gasdermin-mediated pore formation and is characterized by lytic cell death accompanied by IL-1β and IL-18 release. Pyroptosis can elicit potent immunostimulation, yet excessive pyroptosis may also drive detrimental inflammation. Currently, direct evidence for the induction of pyroptosis by standard clinical cryoablation in human tumors remains limited; therefore, it should be regarded as a biologically plausible but underexplored mechanism rather than an established pathway (24).

In this process, secondary biochemical stress releases DAMPs such as ATP and HMGB1. Through the TLR4/MyD88 signaling axis, these molecules activate the NLRP3 inflammasome, drive maturation of pro-IL-1β, and shape an inflammatory microenvironment. The hypoxic microenvironment can induce stable expression of HIF-1α, aggravate mitophagy by upregulating the pro-apoptotic protein BNIP3, and increase vascular permeability by disrupting endothelial junction proteins such as VE-cadherin. Bioactive substances released during this process, including tumor-specific antigens, free nucleic acid fragments (DNA/RNA), heat-shock proteins, CXCL-family chemokines, and proinflammatory cytokines such as IL-6 and TNF-α, together constitute the core components of the cryo-immunogenic microenvironment (16). These components are captured and processed by dendritic cells and presented to T cells through major histocompatibility complexes. Activation of CD8+ cytotoxic T lymphocytes is particularly important, as it stimulates the host immune system to generate specific antitumor immune responses against primary or metastatic tumors (16, 25).

### 4.2 Effector T-cell activation

Relevant studies have confirmed that cryoablation exerts antitumor effects mainly by activating two types of specific T lymphocytes: CD4+ and CD8+ T cells (26, 27). Compared with radiofrequency ablation, cryoablation has unique advantages in inducing antigen-specific immune responses. Although both techniques can trigger CD8+ T-cell responses, cryoablation more prominently activates CD4+ T cells and may therefore have stronger efficacy in tumor immune targeting (28). The main mechanism involves the release of DAMPs induced by cryoinjury, including high-mobility group box 1 (HMGB1), heat-shock protein 70, adenosine triphosphate, and uric acid (29).

These molecules bind to Toll-like receptors (TLRs) on the surface of dendritic cells (DCs). Of particular importance is the interaction between HMGB1 and TLR4, which significantly enhances the antigen-presenting ability of DCs and promotes antigen migration to lymph nodes, thereby effectively activating T cells (23). This connection between innate and adaptive immunity is crucial for establishing durable antitumor immune memory (30). Studies have also shown that in Lewis lung cancer models treated with cryoablation, levels of cytokines such as IL-2 and IFN-γ are significantly increased in the tumor microenvironment. IL-2 binds to its receptor through autocrine and paracrine mechanisms, activates STAT5, MAPK, and mTORC1 signaling, promotes T-cell survival and expansion, and enhances CD8+ T-cell cytotoxicity through IL-2 secreted by CD4+ T cells. IFN-γ directly enhances the cytotoxic functions of CD8+ T cells and natural killer (NK) cells through the JAK–STAT pathway (31, 32). These multilayered immune activation mechanisms together constitute the distinctive antitumor immune effect of cryoablation.

### 4.3 Tumor microenvironment modulation

Immune escape is a key mechanism that enables tumor cells to survive, proliferate, and metastasize *in vivo*. Its essence lies in the ability of tumor cells to evade immune recognition and attack through multiple strategies. The tumor microenvironment plays a crucial role in this process. Enriched immunosuppressive cells, including regulatory T cells (Tregs), myeloid-derived suppressor cells (MDSCs), and M2-type macrophages, secrete inhibitory cytokines such as transforming growth factor-β (TGF-β) and interleukin-10 (IL-10), forming a precise immunoregulatory network that directly weakens the antitumor functions of effector T cells and NK cells. Continuous recruitment and activation of immunosuppressive cells further constructs an immunosuppressive microenvironment favorable to tumor escape.

Cryoablation can disrupt this pathological state and offers an innovative strategy for reversing immune evasion. Its mechanisms are primarily reflected in three aspects. First, cryoablation reduces the number of immunosuppressive cells and decreases programmed death-ligand 1 (PD-L1) expression, thereby blocking the PD-1/PD-L1 pathway (33). Second, cryoablation increases inflammatory myeloid cell infiltration, activates dendritic cells and type I interferon signaling, and promotes effector lymphocyte infiltration at the treatment site and in distant tumors; it may also reduce Tregs or modulate TGF-β signaling (34–36). Third, cryoablation-induced local inflammation and tumor antigen release activate antigen-presenting cells, facilitate effector immune cell infiltration, and establish a positive cycle of immune activation and tumor clearance.

However, these changes are markedly dynamic and bidirectional. Temporally, Treg abundance did not significantly decrease at day 3 after cryoablation in a lung adenocarcinoma study, but did decrease at day 30, suggesting that early and late biopsies may yield different conclusions (37). In terms of ablation parameters, slow freezing in a breast cancer model increased Tregs and metastasis, whereas rapid freezing induced stronger antitumor immunity (16); in the poorly immunogenic B16F10 melanoma model, cryoablation conferred little systemic effector immune benefit despite effective tumour reduction (9, 26, 34). Moreover, adaptive PD-L1 upregulation, MDSC recruitment, M2-like macrophage polarisation, hypoxia, and reparative TGF-β signaling may limit the efficacy of incomplete or suboptimally timed cryoablation. Thus, the post-ablation microenvironment is best understood as a bidirectional, time-dependent state in which pro-immune and immunosuppressive programs coexist. The net outcome depends on tumour genotype and immunogenicity, host factors, baseline immune infiltration, ablation parameters, residual viable tissue, and systemic therapy.

**Figure 1:**
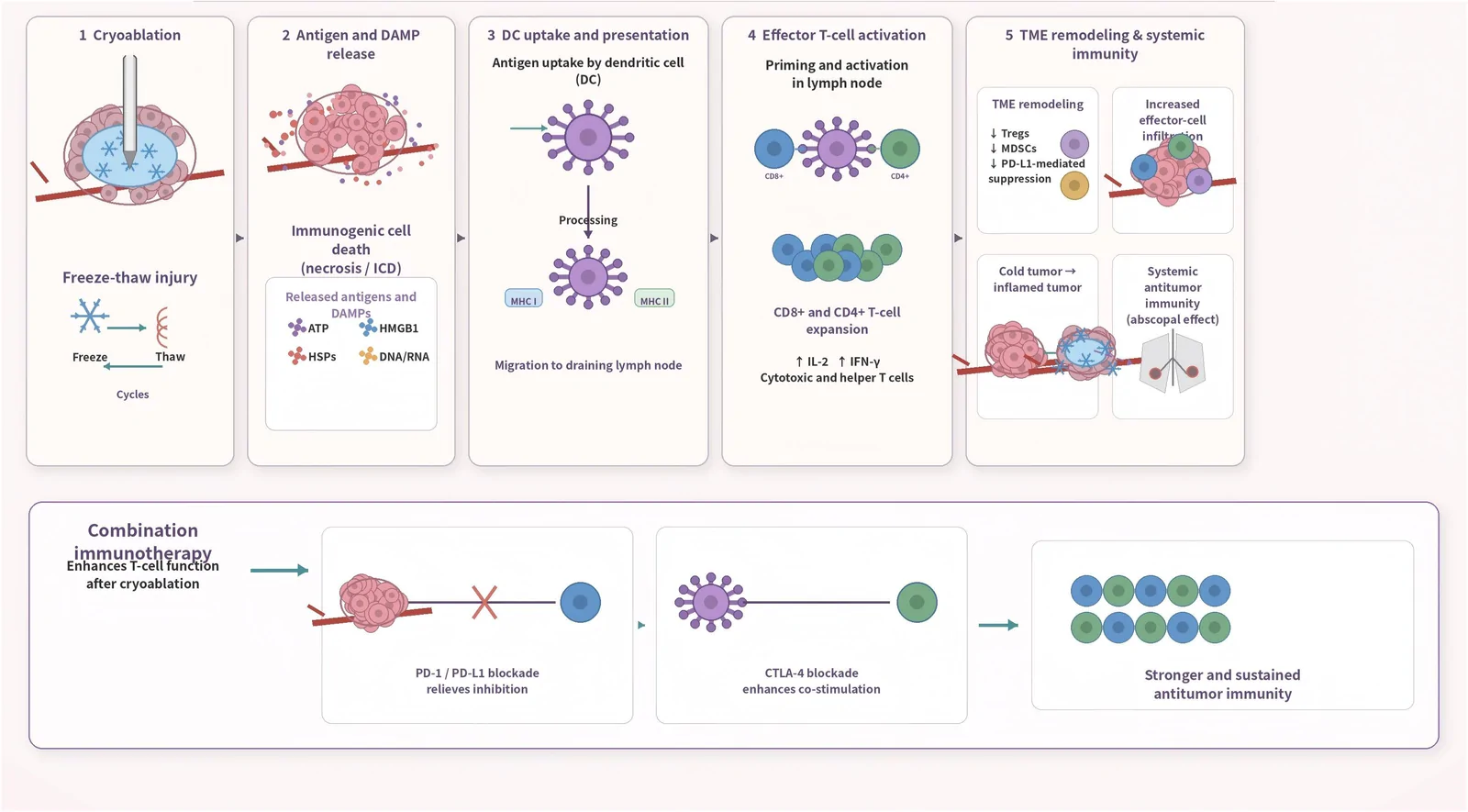
Immunological mechanism of cryoablation-based therapy. Cryoablation induces immunogenic cell death through freeze-thaw injury, releases tumor antigens and DAMPs, promotes dendritic-cell uptake and antigen presentation, and activates CD4+/CD8+ T cells; immune-checkpoint blockade can further strengthen effector T-cell function and systemic antitumor immunity. (DAMP, damage-associated molecular pattern; ICD, immunogenic cell death; DAMPs, damage-associated molecular patterns; ATP, adenosine triphosphate; HMGB1, high mobility group box 1; HSPs, heat shock proteins; DNA, deoxyribonucleic acid; RNA, ribonucleic acid; DC, dendritic cell; MHCI, major histocompatibility complex class I; MHCII, major histocompatibility complex class II; CD8, cluster of differentiation 8; CD4, cluster of differentiation 4; IL-2, interleukin-2; IFN-γ, interferon-gamma; TME, tumor microenvironment; Tregs, regulatory T cells; MDSCs, myeloid-derived suppressor cells; PD-L1, programmed death-ligand 1; PD-1, programmed cell death protein 1; CTLA-4, cytotoxic T-lymphocyte-associated protein 4).

This multitarget immunomodulatory effect, centered on the tumor microenvironment, endows cryoablation with distinctive potential in immunotherapy; however, the current evidence does not support cryoablation as a universal strategy for consistently eliminating Tregs or MDSCs. Thus, the post-ablation microenvironment is best understood as a bidirectional, time-dependent state in which pro-immune and immunosuppressive programs coexist (**Figure 1**).

## 5 Exploration of cryoablation combined with immunotherapy

### 5.1 Initial exploration

The concept of cryoimmunology originated in 1967, when Yantorno first demonstrated that cryoablation could induce specific antibodies in rabbit tissues, with an immune response pattern highly similar to that elicited by autologous tissue homogenates (38). This study suggested that cryoablation releases immunogenic autoantigens capable of breaking immune tolerance, thereby laying the foundation for cryoimmunology.

Subsequent clinical and experimental observations further established the systemic nature of cryoablation-induced immunity. In 1971, Ablin reported spontaneous regression of untreated metastatic lesions in prostate cancer patients following local cryotherapy, providing the first clinical evidence of an abscopal effect (39). Later, Misao and colleagues confirmed this phenomenon in bilateral tumor models, showing that cryoablation of one side significantly inhibited contralateral tumor growth, indicating that the induced immune response is systemic and T-cell mediated (40). Udagawa’s team subsequently demonstrated that cryoablation-induced cytotoxic T lymphocytes (CTLs) exhibited highly specific killing activity exclusively against the homologous tumor, clarifying that the post-ablation immune response is antigen-specific (41). These early mechanistic studies also highlighted the opposing roles of CD8^+^ CTLs and immunosuppressive Tregs, though many underlying pathways remained unexplored, and most evidence was derived from animal models with limited clinical validation.

Building on these findings, subsequent investigations explored strategies to amplify cryo-induced antitumor immunity. Alteber and colleagues showed that intratumoral injection of immature dendritic cells during cryoimmunotherapy induced not only a robust tumor-specific CTL response but also effector memory T cells, which conferred protection against tumor rechallenge and reduced metastasis (42). This prompted the combination of dendritic cells with the immune adjuvant CpG oligodeoxynucleotide (CpG-ODN), which demonstrated enhanced tumor inhibition and improved survival in tumor-bearing mice (43).

### 5.2 Further development

The advent of immune checkpoint inhibitors (ICIs) provided a rational strategy to potentiate and sustain cryoablation-induced antitumor immunity. ICIs function by releasing the brakes on T-cell activation, and unlike many conventional anticancer agents, their inhibitory effect on immune evasion pathways can be more durable (44). Clinically established agents primarily target PD-1/PD-L1 and CTLA-4. PD-1 suppresses effector T-cell function upon binding to its ligands; PD-1/PD-L1 inhibitors block this interaction and restore T-cell activity (45). CTLA-4, which competes with CD28 for CD80/CD86 ligands with higher affinity, downregulates T-cell responses, whereas CTLA-4 inhibitors enhance CD28-mediated costimulation (46, 47).

Preclinical and clinical studies have evaluated this combination strategy. In a prostate cancer mouse model, Waitz and colleagues demonstrated that cryoablation activated T cells, but suppressive signals such as the CTLA-4 pathway limited their activity; addition of anti-CTLA-4 antibody successfully blocked this inhibition, significantly enhancing CD8^+^ T-cell expansion and infiltration, with more pronounced control of secondary tumors (48). In a clinical pilot, Gu and colleagues treated six patients with unresectable NSCLC using cryoablation combined with anti-PD-1 therapy. Paired biopsies from three patients revealed increased CD8^+^ tumor-infiltrating lymphocyte infiltration in target lesions, indicating a shift from immune exclusion to immune activation (35).

From a safety perspective, a retrospective cohort study of patients with advanced solid tumors reported no uncontrollable or unexpected toxicities, and no fatal treatment-related events such as severe bleeding or pneumonia (49). However, combination of CTLA-4 and PD-1 blockade, while significantly improving objective response rates and overall survival compared with single-agent therapy, is associated with a higher incidence of immune-related adverse events, particularly severe gastrointestinal toxicity and hepatotoxicity (50).

### 5.3 Recent progress

Recent investigations have further elucidated the immunological mechanisms underlying cryoablation and explored novel combinatorial strategies. Building on the established evidence that cryoablation enhances tumor antigen immunogenicity, promotes antigen-presenting cell maturation, and increases effector T-cell numbers (51), Gu and colleagues observed in lung cancer mouse models that ablation remodeled the tumor immune microenvironment, with increased expression of type I interferon-dependent antitumor immune components and activation of the STING signaling pathway, as evidenced by upregulated phosphorylation of STING and TBK1 (35). Given the known synergy between immune checkpoint inhibitors and type I interferons, these findings suggest that cryoablation may enhance ICI efficacy through STING-dependent type I IFN responses.

Emerging technological innovations have also expanded the therapeutic landscape. Nanocatalyst-assisted cryoablation has been shown to promote ice formation and enhance cryotoxicity, thereby stimulating antitumor inflammatory pathways, including necroptosis, and improving immunogenicity (22). However, clinical evidence for this approach remains scarce, and safety data are insufficient, warranting large-scale trials. Cryo-thermal therapy (CTT), which integrates precooling with ablation, represents another advance. Its core mechanism involves an early neutrophil response that releases reactive oxygen species to activate monocytes, initiating a cascade of immune activation and upregulating cytotoxic molecules such as IFN-γ and granzyme B in T and NK cells. CTT-induced neutrophil migration has been shown to significantly enhance antimetastatic efficacy in preclinical models, an effect attributed to neutrophil-mediated remodeling of the immune microenvironment (52).

A recent study from Shanghai Pulmonary Hospital demonstrated that cryoablation effectively promotes double-stranded DNA release from tumor cells, activating the cGAS–STING pathway in macrophages. This process induces abundant infiltration of CXCL10^+^ macrophages, which recruit CXCR3^+^ T cells into the tumor microenvironment (36). Notably, compared with thermal ablation, cryoablation combined with anti-PD-1 therapy induced a stronger systemic antitumor immune response, converting immunologically cold tumors into hot ones—offering a clear clinical strategy to overcome immunotherapy resistance in NSCLC.

## 6 Applications of cryoablation combined with immunotherapy

### 6.1 Combination with immune checkpoint inhibitors

Cryoablation combined with immune checkpoint inhibitors has become one of the most promising combination strategies in tumor therapy. The major immune checkpoints include CTLA-4 and PD-1. Representative inhibitors include ipilimumab (anti-CTLA-4), pembrolizumab, nivolumab, sintilimab, and atezolizumab (anti-PD-1/PD-L1). Synergy between CTLA-4 or PD-1 inhibitors and cryoablation has been confirmed in animal experiments. In a prostate cancer model, cryoablation combined with anti-CTLA-4 antibody significantly prolonged mouse survival and reduced lethality to one-quarter (48). In a murine model of renal cell carcinoma, the combination of cryoablation and PD-1 blockade effectively amplified the anti-tumor immune response initially triggered by cryoablation, significantly elevating the infiltration level of CD8^+^ tumor-infiltrating lymphocytes, alongside marked upregulation of the key anti-tumor effector molecules IFN-γ and granzyme B (53).

Clinical studies have further confirmed its advantages. In patients with stage IIB–IV NSCLC treated with cryoablation combined with PD-1 inhibitors, total CD4+ cells, CD8+ T cells, NK cells, and serum IFN-γ levels increased compared with the monotherapy group. Tumor cells and tumor markers decreased, without an increased risk of adverse reactions (44). In patients with melanoma liver metastasis (n = 15), cryoablation combined with pembrolizumab prolonged median progression-free survival to 8.2 months compared with 4. l months in the monotherapy group, and hepatic progression-free survival to 10.5 months compared with 5.3 months. Serum IL-6 increased 2.3-fold and the proportion of NK cells increased l.8-fold (54).

Mechanistic studies suggest that this combination induces tumor antigen release through cryoablation, breaks immune tolerance, and simultaneously relieves T-cell functional suppression through immune checkpoint inhibition, forming a synergistic chain of antigen release, immune activation, and enhanced effector function. A randomized phase II trial analyzing outcomes after anti-PD-1/Ll treatment in advanced NSCLC divided patients into ablation plus immunotherapy and immunotherapy-alone groups. The addition of ablation improved tolerability and prolonged survival after anti-PD-1/Ll therapy in advanced NSCLC, while cryoablation showed potentially greater survival benefit than thermal ablation (55). Systemic immune activation has now been observed in melanoma, breast cancer, lung cancer, prostate cancer, liver cancer, and other solid tumors, providing a new model for comprehensive tumor treatment.

### 6.2 Combination with adoptive immunotherapy

Adoptive cell therapies, including NK cells, DCs, cytokine-induced killer (CIK) cells, and T lymphocytes, exert antitumor effects by enhancing immune responses (12). NK cells directly or indirectly inhibit tumors in early tumor defense through cytokine secretion. DCs, as key antigen-presenting cells, can activate specific T cells, and their efficacy can be improved through cryoablation combined with DC vaccines. Combination of adoptive cells such as CIK cells with cryoablation has been applied in colorectal liver metastases, lung cancer, and other tumors, improving remission rates, enhancing immune function, and improving quality of life (56).

Adoptive cell therapy enhances antitumor capacity by expanding and functionally modifying autologous immune cells *in vitro*, such as CAR-T and TCR-T cells. Tumor antigens released by cryoablation can provide additional targets, and the combination of the two can significantly improve efficacy. Related studies in hepatocellular carcinoma and renal cell carcinoma have shown that cryoablation combined with adoptive cell therapy can prolong survival and improve quality of life. Cryoablation combined with DCs, NK cells, CIK cells, and other adoptive cellular therapies has also shown significant antitumor potential in preclinical and clinical studies through mechanisms such as optimized antigen presentation and enhanced effector cell activity, providing multiple feasible new routes for immunotherapy of solid tumors (57).

### 6.3 Combination with toll-like receptor agonists

Toll-like receptors (TLRs), as key pattern-recognition molecules on the surface of macrophages and DCs, specifically recognize pathogen-associated molecular patterns such as CpG-ODN, trigger DC maturation, and drive migration to draining lymph nodes (58). In this process, mature DCs efficiently present tumor antigen peptides and activate T-cell-mediated specific antitumor immune responses. In contrast, non-activated DCs induce immune tolerance toward tumors.

Based on this immunoregulatory mechanism, TLR agonists, including CpG-ODN and imiquimod, can act as efficient immune adjuvants and significantly improve antitumor efficacy when combined with cryoablation through multiple synergistic effects. Animal experiments have shown that in lung cancer mouse models, combination therapy significantly suppresses tumor growth, lowers distant metastasis rates, and prolongs survival (59). Melanoma model studies further confirmed that this combination enabled 90% of mice to reject tumor rechallenge, whereas cryosurgery alone failed to prevent tumor grafting in 70% of cases (60). In patients with highly recurrent basal cell carcinoma, the tumor recurrence rate within 18 months after combination therapy was as low as 5%.

At the molecular level, TLR signaling can induce metabolic reprogramming of innate immune cells and promote secretion of key inflammatory mediators such as type I interferons (IFN-α/β), thereby strengthening the intensity and durability of T-cell immune responses. Notably, tumor vaccines, such as whole-cell vaccines and peptide vaccines, can also induce specific antitumor immune effects by activating CD8+ cytotoxic T lymphocytes and CD4+ helper T cells. These immune interventions and the TLR agonist/cryoablation combination jointly construct a multidimensional therapeutic network for enhancing antitumor immune responses through complementary mechanisms.

### 6.4 Combination with tumor vaccines

Tumor vaccines activate and expand T cells by delivering tumor-specific antigens, thereby achieving precise killing of tumor cells. Cryoablation not only releases large quantities of immunogenic tumor antigens but also synchronously activates DCs, providing vaccines with richer antigenic targets and strengthening T-cell-mediated immune effects. Together, these approaches construct a more efficient antitumor immune network (47). DCs are the core target cells in the functional pathway of cancer vaccines. Their degree of infiltration and functional maturation in the tumor microenvironment are key determinants of the final immune activation efficacy of cancer vaccines (61).

Cryoablation can generate a patient-specific antigen depot, creating a rationale for whole-tumor, dendritic-cell, peptide, or nanomaterial-assisted vaccination. In a triple-negative breast cancer model, cryoablation combined with Meriva reduced IL-6-mediated immunosuppression and delayed lung metastasis (62). Lymph-node-targeting nanovaccines and liquid-metal nanoplatforms can capture cryo-released antigens, promote cross-presentation, and improve distant tumor control in animal models (63, 64). However, these findings should be regarded as preclinical proof of concept. Nanoparticle composition, biodegradability, batch reproducibility, antigen-loading variability, pharmacokinetics, manufacturing scale, and long-term toxicity remain unresolved. Currently, no mature clinical evidence demonstrates that cryoablation-generated nanovaccines improve patient survival. Therefore, claims of immediate clinical applicability would be premature; the priority should be early-phase safety, biodistribution, and immune-pharmacodynamic studies. Future research should focus on translating these promising preclinical strategies into well-designed clinical trials to establish their feasibility and therapeutic value.

### 6.5 Relieving immunosuppression after cryoablation

Beyond the direct activation of innate and adaptive immunity by cryoablation, a second distinct strategy to amplify systemic antitumor immunity is to selectively block the dominant immunosuppressive pathways that are specifically upregulated in the post-cryoablative tumour microenvironment. Preclinical studies have demonstrated that targeting activated leukocyte cell adhesion molecule (ALCAM) in the post-cryoablation niche can effectively restore immune cell trafficking and potentiate systemic antitumor immunity (65). Anti-angiogenic agents, exemplified by lenvatinib in the CASTLE-01 trial, implement this concept by normalizing the dysfunctional tumour vasculature, mitigating VEGF-driven accumulation of myeloid-derived suppressor cells, and subsequently promoting effector lymphocyte infiltration into the tumour parenchyma (66). As an alternative physical intervention, sequential cryo-heat ablation has also been reported to trigger neutrophil-derived reactive oxygen species (ROS) cascades, which further reprogram and activate circulating monocytes to reinforce systemic antitumor immune responses (52).

Beyond these proof-of-concept observations, a broader panel of downstream immunosuppressive targets has been systematically validated in multiple preclinical tumour models, all of which have been shown to be significantly upregulated in the post-cryoablation TME rather than in untreated tumors. These targets include TGF-β (67), IL-6, VEGF, CXCR2-dependent myeloid cell recruitment, CSF1R-dependent tumour-associated macrophage polarization (68), the adenosine signaling axis, and regulatory T-cell (Treg) depletion. Such interventions are expected to achieve optimal clinical benefit specifically in patients whose baseline or post-ablation biopsies exhibit a myeloid-dominant immunosuppressive landscape, insufficient T-cell infiltration, elevated TGF-β signaling, or adaptive immune checkpoint upregulation. Nevertheless, these immunomodulatory agents carry non-negligible procedure-specific safety risks: they may impair post-ablation tissue repair, increase the risk of cryo-needle tract bleeding, or exacerbate hypertension and immune-related adverse events, mandating rigorous procedure-aligned safety evaluation before widespread clinical translation.

### 6.6 Evidence across tumor types

The preceding subsections organized the evidence by the immunotherapeutic modality combined with cryoablation. Given that the depth and robustness of these data vary considerably across oncological indications, the present subsection re-examines the relevant literature from a disease-centric perspective instead. This rearrangement helps clinicians managing a specific tumor type to more readily determine which level of evidence pertains to their practice. Key illustrative studies are compiled in **Table 1**, while **Table 2** lists the ongoing registered trials that are likely to alter the treatment landscape in the near term.

**Table 1:** Representative clinical and translational evidence for cryoablation combined with immunotherapy, by tumor type and evidence tier.

Tumor type/context	Evidence tier	Combination and design	Sample	Main signal	Key safety/limitations	Ref.
NSCLC, oligo-residual after anti-PD-1/L1	1 (randomized)	Ablation + continued ICI vs ICI maintenance; randomized phase II	65 randomized; 62 analyzed	Median PFS 26.7 vs 11.7 months (HR 0.213, p < 0.001)	Closed before planned enrollment; cryoablation in only 13/42 ablated patients; 1 grade 3 pneumothorax	(55)
Advanced NSCLC, stage IIIB–IV	2	Argon–helium cryoablation + camrelizumab vs PD-1 inhibitor + platinum chemotherapy; single-center randomized	60	Median PFS 9.6 vs 8.3 months (p=0.005); OS advantage; ORR 73.3% vs 53.3% (p=0.108, NS)	Single center; open label; 1-year follow-up; no tumor microenvironment analysis	(70)
Advanced NSCLC, untreated, PD-L1 ≥50%	2	Cryoactivation + pembrolizumab; single-arm pilot	8	ORR 25%; CD8 recruitment in patients with benefit	No benefit vs historical ICI monotherapy; no grade 3–4 events	(69)
Advanced/metastatic intrahepatic cholangiocarcinoma	2	Cryoablation → sintilimab + lenvatinib; single-arm phase II	28	ORR 75.0%; DCR 100%; median PFS 16.8 months; median OS 25.4 months	Three-component regimen prevents attribution; single arm; grade ≥3 TRAE 36%	(71)
Advanced hepatocellular carcinoma	2	Cryoablation ± allogeneic NK cells; prospective non-randomized	61	Median PFS 9.1 vs 7.6 months (p=0.0107); further gain with repeated infusions	Non-randomized; no OS reported; median follow-up 8.7 months	(72)
Melanoma after ICI progression	2	Cryoablation + continued ICI; phase II	17 evaluable	ORR 23.5%, DCR 41.2% in non-ablated lesions; 1-year OS 53%	Small, non-randomized; median PFS 59 days; minimal post-ablation tissue	(75)
Melanoma liver metastases	2–3	Cryoablation + transarterial pembrolizumab (cohort); cryoablation during PD-1 blockade (retrospective)	15; 45	ORR 26.7%; ablating lesions ≤30 mm: response 42.9% vs 12.5% (p = 0.022), OS 30.5 vs 14.2 months (p = 0.045)	Ambispective/retrospective; no concurrent control; subgroup-derived cut-points	(54, 76)
Oligometastatic hormone-sensitive prostate cancer	2	Prostate cryotherapy + pembrolizumab + androgen deprivation; single-arm pilot	12	PSA <0.6 ng/mL at 1 year in 42%; median PFS 14 months	Concurrent androgen deprivation prevents attribution; all AEs grade ≤2	(79)
Metastatic castration-resistant prostate cancer	2	Cryoablation + autologous dendritic cells ± ipilimumab or pembrolizumab; phase I	18	Durable benefit 33%; median PFS 10.5 months, median OS 40.7 months; TCR repertoire remodeling	Phase I, no control; checkpoint subgroups n=6 and n=3; heterogeneous prior therapy	(80)
Metastatic renal cell carcinoma	2	Tremelimumab ± cryoablation; randomized pilot	29	CD3+ (p = 0.002) and CD8+ (p = 0.009) infiltration increased in clear-cell tumors; ORR 3.4%	Immune activation without clinical advantage; grade ≥3 TRAE 55%; histologic heterogeneity	(83)
Localized/early renal cell carcinoma	2	Cryoablation vs surgery; prospective immunomonitoring with TCR sequencing	22; 25	Oligoclonal T-cell expansion; increased activated and central-memory CD8+ at 3 months	No efficacy endpoints; small; single post-treatment time point	(84, 85)
Early breast cancer	2	Preoperative cryoablation and/or ipilimumab; cryoablation + ipilimumab and nivolumab; pilots	19 and 5	Effector: Treg ratio increased (p = 0.05); repertoire diversification in 100% of cryoablated tumors (p = 0.005)	Not powered for recurrence or survival; 1 grade 4 hepatic toxicity in the five-patient dual-blockade pilot	(86–88)
Early breast cancer (procedural platform)	2	Cryoablation without excision; prospective multicenter single arm	194	5-year ipsilateral recurrence 4.3%; breast cancer survival 96.7%	No immunologic endpoints; highly selected low-risk population; no randomized surgical comparator	(93)
Metastatic soft-tissue sarcoma	3	Cryoablation during ipilimumab and nivolumab; retrospective	16 patients, 27 procedures	Local complete response wherever complete ablation intended; median OS 14.1 months	Retrospective; median PFS 2.3 months; 5 pneumothoraces; heterogeneous subtypes	(97)
Metastatic and locally advanced pancreatic cancer	3	Cryoablation + cellular immunotherapy (retrospective); cryoablation alone (single arm)	106; 24	Median OS 13 vs 7 months (cryo-immunotherapy vs cryoablation); median OS 16.8 months in locally advanced disease	Retrospective; severe allocation bias; older treatment era; immune markers unspecified	(57, 94)
Colorectal cancer	3 (preclinical) and technical	Adjunctive partial cryoablation + dual checkpoint blockade (MC-38/CT-26); three-dimensional margin analysis of pulmonary metastases	Murine; 38 patients/76 lesions	60-day survival 79% vs 61%; distant-tumor activated CD8 + 30.3% vs 5.9% (p = 0.006); margin >5 mm HR 0.03 (p = 0.005)	No clinical study of cryoablation + immunotherapy exists in colorectal cancer	(95, 96)
Bone metastasis	3 (preclinical)	Cryoablation ± anti-PD-1; murine bone-metastasis model	8 per group	Abscopal tumor shrinkage; tumor-specific ELISPOT 107.3 vs 31.6 spots (p<0.01)	Murine; single cell line; no human trial exists	(98)
HCC/NSCLC/other, adoptive cellular therapy	3	Cryoablation + NK, CIK or mixed cellular therapy; small cohorts	Variable	Reported immune, quality-of-life or survival signals	Non-standard cell products; historical controls; older treatment era	(10, 57, 72, 73)

ICI, immune checkpoint inhibitor; ORR, objective response rate; DCR, disease control rate; PFS, progression-free survival; OS, overall survival; TRAE, treatment-related adverse event; TCR, T-cell receptor; PSA, prostate-specific antigen. Evidence tier: 1, randomized clinical data; 2, prospective single-arm or pilot data with immune correlates; 3, retrospective series or preclinical models.

**Table 2:** Registered ongoing trials of cryoablation combined with immunotherapy, by tumor type.

Tumor type	Trial (registry identifier)	Phase	Design	Planned N	Status
Metastatic lung adenocarcinoma, first line	CRYOMUNE (NCT04339218)	III	Cryoablation added or not to pembrolizumab + pemetrexed–carboplatin; randomized 1:1; primary endpoint 1-year OS	214	Recruiting
Advanced non-squamous NSCLC, first line	NCT06483009	II	Sintilimab + chemotherapy with cryoablation; single arm; primary endpoint PFS	20	Not yet recruiting
Hepatocellular carcinoma	NCT06530784	II	Cryoablation + anti-PD-1 antibody + bevacizumab; single arm, open label	36	Not yet recruiting
Intrahepatic cholangiocarcinoma, first line	CASTLE-ICC-Chemo-free (NCT05835245)	II	Cryoablation + sintilimab + lenvatinib, chemotherapy-free	30	Recruiting
Melanoma resistant to PD-1 blockade	NCT05779423	II	Cryoablation + ipilimumab + nivolumab	37	Recruiting
Early-stage triple-negative breast cancer	LOGIC (NCT05806385)	I/II	Standard care vs cryoablation vs cryoablation + pembrolizumab; primary endpoint pathologic complete response	36	Not yet recruiting
High-risk non-muscle-invasive bladder cancer	NCT07204132	Not applicable	Intracavitary cryoablation + anti-PD-1	180	Recruiting

PFS, progression-free survival; OS, overall survival; PD-1, programmed death-1; NSCLC, non-small cell lung cancer.

#### 6.6.1 Non-small cell lung cancer

Studies in NSCLC provide the only randomized evidence that adding local ablation to ongoing checkpoint blockade improves outcomes. In the BOOSTER trial, 65 patients with advanced NSCLC and oligo-residual disease after anti-PD-1/PD-L1 therapy were randomized 2:1 to ablation plus continued immunotherapy or immunotherapy alone; median PFS was 26.7 versus 11.7 months (HR 0.213, p<0.001) (55). Two caveats apply: the trial closed early, and only 13 of 42 ablated patients received cryoablation (the remainder microwave or radiofrequency). BOOSTER thus supports ablation as a class, not cryoablation specifically. Dedicated prospective data on cryoablation–immunotherapy combinations remain scarce. The only such study to date, CRYOVATE, enrolled eight treatment-naive PD-L1 ≥50% patients, reporting a 25% ORR and no grade 3–4 events, but no benefit over historical ICI monotherapy (69). A single-center randomized study (n=60, stage IIIB–IV) comparing cryoablation plus camrelizumab versus PD-1 inhibitor plus chemotherapy reported longer median PFS (9.6 vs. 8.3 months, p=0.005) and OS benefit, though ORR difference was not significant (73.3% vs. 53.3%, p=0.108) (70). Mechanistic data are more definitive: cryoablation releases tumor DNA that activates the macrophage cGAS–STING–CXCL10 axis and recruits CXCR3+ T cells more efficiently than thermal ablation (36), with type I IFN signaling required for antitumor immunity (35). The phase III CRYOMUNE trial (NCT04339218) is testing cryoablation plus first-line pembrolizumab–chemotherapy and will provide the first definitive test of the strategy.

#### 6.6.2 Hepatocellular carcinoma and biliary tract cancer

In the field of liver oncology, the strongest prospective signal currently comes from biliary rather than hepatocellular malignancy. In the single-arm phase II CASTLE-01 trial, 28 patients with chemotherapy-refractory advanced or metastatic intrahepatic cholangiocarcinoma received cryoablation followed by sintilimab and lenvatinib, achieving an objective response rate of 75.0% (95% CI 59–91), a disease control rate of 100%, median progression-free survival of 16.8 months and median overall survival of 25.4 months, with grade ≥3 treatment-related events in 36% and no treatment-related deaths (71). Because all three components were administered together, the independent contribution of cryoablation cannot be isolated. For hepatocellular carcinoma (HCC) itself, the best prospective data involve adoptive cells rather than immune checkpoint inhibitors: among 61 patients with advanced HCC treated with cryoablation with or without allogeneic natural killer cell infusion, median progression-free survival was 9.1 versus 7.6 months (p=0.0107), with additional PFS prolongation observed in patients receiving repeated infusions, although the study was non-randomized and reported no overall survival data (72). Retrospective cryo-immunotherapy series report substantially longer survival than cryoablation alone (median overall survival 32 versus 17.5 months) but are subject to severe allocation bias (73). The HCC literature also contains an explicit cautionary finding: among 141 patients with HBV-related HCC, of whom 109 underwent cryoablation, elevated circulating PD-L1 was an independent predictor of shorter overall and tumor-free survival, indicating that ablation can install adaptive resistance as well as prime immunity (74). Taken together, hepatobiliary evidence is prospective but not randomized, and it is weaker in HCC than the frequent “NSCLC–HCC” pairing in the literature would suggest.

#### 6.6.3 Melanoma

Melanoma provides the only prospective phase II trial of cryoablation added to checkpoint blockade after immunotherapy progression. Among 17 evaluable patients with checkpoint-refractory metastatic melanoma, cryoablation with continued immune checkpoint inhibition produced an objective response rate of 23.5% (95% CI 6.8–49.9) and a disease control rate of 41.2% in non-ablated lesions, with median progression-free survival of 59 days, one-year overall survival of 53%, and no new grade ≥3 immune-related events after ablation (75). In liver-metastatic disease, cryoablation combined with transarterial infusion of pembrolizumab was feasible in 15 patients, with an objective response rate of 26.7%, a significant increase in circulating natural killer cells and no grade 3–4 events (54). A subsequent retrospective cohort of 45 patients receiving cryoablation during PD-1 blockade identified lesion selection as a determinant of the abscopal effect: ablation of an intrahepatic lesion ≤30 mm was associated with a response rate of 42.9% versus 12.5% (p=0.022) and overall survival of 30.5 versus 14.2 months (p=0.045), whereas ablating lesions larger than 3 cm produced greater increases in neutrophil-to-lymphocyte ratio and interleukin-6 (76). Preclinically, cryoablation loads dendritic cells in draining lymph nodes approximately twice as efficiently as radiofrequency ablation and, when combined with CTLA-4 blockade or regulatory T-cell depletion, protects against tumor rechallenge (77); local cryosurgery combined with imiquimod similarly enhanced systemic immunity (60). Melanoma therefore supplies the clearest demonstration that lesion size and lesion choice—not merely the decision to ablate—govern systemic benefit, a principle that is directly transferable to other tumor types.

#### 6.6.4 Prostate cancer

Prostate cancer is where cryoimmunology began: the early report of regression of untreated metastases after local cryotherapy, remains one of the first observations of an abscopal effect (39). The field retains an unusually complete preclinical-to-clinical chain. In the TRAMP-C2 model, cryoablation plus anti-CTLA-4 caused rejection of secondary tumors in 4 of 9 mice versus none in any control arm, and CD8^+^ T-cell depletion significantly reduced the survival benefit, indicating a CD8-dependent mechanism (48). In the hormone-sensitive Myc-CaP model, cryoablation plus anti-CTLA-4 delayed distant tumor growth by 14.8 days (p = 0.0006) and reduced mortality approximately four-fold, whereas adding anti-PD-1 to cryoablation conferred no benefit over either agent alone—a dissociation between checkpoint targets not yet demonstrated in NSCLC or hepatocellular carcinoma (78). Clinically, a single-arm pilot of whole-prostate cryotherapy with pembrolizumab and androgen-deprivation therapy in 12 men with newly diagnosed oligometastatic hormone-sensitive disease reported prostate-specific antigen below 0.6 ng/mL at one year in 42% and median PFS of 14 months, with all adverse events grade ≤2, although concurrent androgen deprivation prevents attribution to the immunologic component (79). A phase I trial of dendritic-cell-based cryoimmunotherapy in 18 men with metastatic castration-resistant disease, with intratumoral ipilimumab or intravenous pembrolizumab added in later cohorts, showed no dose-limiting toxicity, durable clinical benefit in 33%, median PFS of 10.5 months and median OS of 40.7 months, with measurable TCR repertoire remodeling (80). Earlier work using GM-CSF reported a median PSA decline of 69.4% and time to progression of 18 months in 12 men (81), and a randomized pilot (n=20) demonstrated increased cancer-antigen-related antibody at 4 and 12 weeks (p<0.05) (82). The prostate literature is thus mechanistically rich and consistently safe, but every clinical study remains small, single-arm, or underpowered.

#### 6.6.5 Renal cell carcinoma

Renal cell carcinoma provides the field’s most instructive dissociation between immunological and clinical endpoints. In a randomized pilot of tremelimumab with or without cryoablation in 29 patients with metastatic disease, the combination significantly increased intratumoral CD3^+^ (p = 0.002) and CD8^+^ (p = 0.009) infiltration and induced tertiary lymphoid structures in clear-cell renal tumors, yet the overall objective response rate was 3.4% and neither progression-free nor overall survival favored the cryoablation arm, with grade ≥3 treatment-related adverse events in 55% (83). Two prospective translational studies corroborate the immunological readout without addressing efficacy: paired biopsies obtained before and three months after cryoablation in 22 patients demonstrated significant oligoclonal T-cell expansion with elevated CD8, granzyme A and CD8/FOXP3 ratios (84), and longitudinal flow cytometry in 25 patients showed that cryoablation, unlike nephrectomy, increased activated and central-memory CD8^+^ subsets at three months without the acute postoperative lymphopenia seen after surgery (85). Preclinically, PD-1 blockade amplifies cryoablation-induced CD8^+^ tumor infiltration with upregulation of interferon-γ and granzyme B (53). The lesson from renal cell carcinoma is that immune activation is a necessary but clearly insufficient surrogate: a trial powered on immune endpoints alone can be positive while the clinical result is neutral, which is precisely why the biomarker framework in Section 8.2 insists that candidate markers be evaluated as interaction markers rather than as evidence of benefit in themselves.

#### 6.6.6 Breast cancer

Breast cancer offers the most detailed peri-ablation immune characterization available in humans. In a pilot study of preoperative single-dose ipilimumab and/or cryoablation in 19 women with early-stage disease, the combination significantly increased the intratumoral Ki67^+^ effector-to-regulatory T-cell ratio relative to either monotherapy (p=0.05) (86). TCR sequencing showed that cryoablation, with or without ipilimumab, shifted the intratumoral repertoire toward polyclonality (median clonality change −0.04, p = 0.005), whereas ipilimumab principally increased T-cell density—indicating that ablation and checkpoint blockade act on different axes of the same response (87). A subsequent pilot combining cryoablation with ipilimumab and nivolumab in five women reported increased T-cell activation and reduced CD4+PD-1high cells, but one grade 4 hepatic toxicity among five patients cautions against dual checkpoint blockade in the perioperative setting (88). Preclinical work supports an abscopal mechanism: cryoablation of a primary triple-negative tumor increased CD8^+^ infiltration and granzyme B in the contralateral untreated tumor and abrogated local recurrence and pulmonary metastasis seen after resection (89); it also increased natural killer cell frequency in abscopal tumors and migratory conventional type 1 dendritic cells in the spleen (90). Rapid freezing again generates stronger tumor-specific immunity than slow freezing (91, 92). Separately, the ICE3 trial demonstrated that cryoablation without surgical excision can control selected low-risk early breast cancers, with a five-year ipsilateral breast tumor recurrence rate of 4.3% in 194 women, providing the procedural foundation for ongoing randomized combination trials such as LOGIC (NCT05806385) (93).

#### 6.6.7 Other solid tumors and current evidence gaps

Evidence in the remaining tumor types is retrospective, preclinical, or absent. In metastatic pancreatic cancer, a retrospective series of 106 patients reported median overall survival of 13 months with comprehensive cryoablation plus cellular immunotherapy, versus 7 months with cryoablation alone, 5 months with immunotherapy alone and 3.5 months with chemotherapy (57); a more recent single-arm series of 24 patients with locally advanced disease reported median overall survival of 16.8 months with no perioperative deaths (94). In colorectal cancer, no clinical study has been published; preclinical evidence shows that partial cryoablation added to dual checkpoint blockade improves 60-day survival in MC-38 (79% vs 61%) and CT-26 (57% vs 35%, p = 0.04) models, with increased activated CD8^+^ T cells in distant tumors (30.3% vs 5.9%, p = 0.006) (95); technical work has established that a 3D minimal margin >5 mm independently predicts local control of pulmonary colorectal metastases (HR 0.03, p = 0.005) (96). In bone and soft-tissue sarcoma, a retrospective series of 16 patients undergoing cryoablation during ipilimumab–nivolumab reported complete local response where intended, median overall survival of 14.1 months, but median progression-free survival of only 2.3 months (97); in a murine bone-metastasis model, cryoablation markedly increased tumor-specific ELISPOT responses versus controls (107.3 vs 31.6 spots, p<0.01), further augmented by anti-PD-1 (123.3 spots), with significant abscopal shrinkage in the combination arm (98). Cryoablation monotherapy achieved a 12-month non-progression rate of 86% in 50 patients with progressing desmoid tumors (99). No published clinical study exists for head and neck, gynecologic, esophageal, or hematologic malignancies; bladder studies remain small and unreported (NCT04701918, n=9 completed; NCT07204132, recruiting). Naming these gaps is as important as summarizing the positive data, because absence of evidence in a tumor type is frequently misread as generalizability from another.

#### 6.6.8 An evidence hierarchy across tumor types

Read across diseases, four tiers emerge. Tier 1, randomized evidence powered for clinical endpoints, exists only in NSCLC, and even there the pivotal trial tested ablation as a class, with cryoablation used in 13 of 42 ablated patients (55); the randomized studies in renal cell carcinoma and prostate cancer were pilots sized for immunological rather than efficacy endpoints (82, 83). Tier 2, prospective single-arm or pilot evidence accompanied by immune correlates, comprises intrahepatic cholangiocarcinoma, melanoma, prostate cancer, renal cell carcinoma and breast cancer, in which feasibility and immune activation are reproducible but efficacy is either unproven or, in renal cell carcinoma, explicitly not demonstrated (71, 75, 79, 80, 83–86, 88). Tier 3, retrospective series and preclinical models, covers hepatocellular combination therapy, pancreatic, colorectal and bone or soft-tissue disease (57, 73, 94, 95, 97, 98). Tier 4, no clinical data at all, includes head and neck, gynecologic, esophageal and most remaining solid tumors. Two conclusions follow. First, the mechanistic rationale is genuinely pan-tumor: the immunological findings summarized in Section 4 have been reproduced in every model system in which they have been sought, and the constraint on generalization is the distribution of clinical trials rather than the biology. Second, the practical recommendations offered in Section 8 are therefore stated at the level of disease state—oligo-residual, oligoprogressive, immune-cold, myeloid-rich—rather than at the level of histology, because disease state transfers across tumor types whereas trial results do not.

### 6.7 Structured comparison of major strategies

Direct comparisons across different studies are inherently limited by substantial heterogeneity in tumor types, disease burden, response criteria, line of therapy, completeness of ablation, and the availability of non-ablated measurable lesions for efficacy assessment. To date, no adequately powered, large-scale head-to-head randomized controlled trial has been conducted to establish a universally applicable ranking among combination strategies—including immune checkpoint inhibitors (ICIs), adoptive cell therapy, immune agonists, tumor vaccines, or immunosuppression-relieving agents. Among all combination approaches, ICI-based regimens currently possess the highest level of clinical maturity, with a randomized phase II trial having demonstrated a significant progression-free survival benefit in selected NSCLC patients. In contrast, although the other strategies are mechanistically compelling and supported by preclinical or early-phase clinical data, their evidence certainty is substantially lower, with most studies being small, uncontrolled, or exploratory in nature. **Table 1** and **Table 3** summarize representative clinical evidence, safety profiles, and translational readiness for these major strategies.

**Table 3:** Comparison of major cryoablation-immunotherapy strategies.

Combination strategy	Mechanistic objective	Most plausible setting	Clinical maturity	Main safety issues	Ref.
PD-1/PD-L1 blockade	Rescue exhausted T cells after antigen release	Oligoresidual/oligoprogressive disease on ICI; selected resistant lesions	Highest; randomized phase II evidence for local ablation + ICI in NSCLC	ICI toxicity plus organ-specific procedural complications	(55)
CTLA-4 blockade	Enhance priming and effector/Treg ratio	Immune-priming deficiency; trial settings	Preclinical and small pilot clinical data	Higher rates of colitis, hepatitis, endocrinopathy	(48, 50)
Adoptive NK/DC/CIK/T cells	Add effector cells or antigen presentation	Low endogenous effector-cell number/function	Small heterogeneous cohorts	Infusion reactions, manufacturing variability, cell- specific toxicities	(10, 57)
TLR/STING/innat e agonists	Provide co-stimulation and innate sensing	Antigen release with inadequate APC activation	Strong preclinical; early translational	Cytokine inflammation, local tissue injury	(42, 58)
Vaccines/nanovac cines	Capture and cross-present autologous antigens	Personalized *in situ* vaccination research	Predominantly animal studies	Nanomaterial toxicity, biodistribution, manufacturing	(63, 64)
Suppression-relieving agents	Reduce TGF- β/VEGF/myeloid/Treg barriers	Myeloid-rich, excluded, angiogenic, or fibrotic tumors	Preclinical plus selected multi-agent clinical regimens	Bleeding, hypertension, wound-healing and overlapping immune toxicity	(66–68)

APC, antigen-presenting cell; CIK, cytokine-induced killer; CTLA-4, cytotoxic T-lymphocyte-associated protein 4; DC, dendritic cell; ICI, immune checkpoint inhibitor; NK, natural killer; NSCLC, non-small cell lung cancer; PD-1, programmed death-1; PD-L1, programmed death-ligand 1; STING, stimulator of interferon genes; TGF-β, transforming growth factor-β; TLR, Toll-like receptor; Treg, regulatory T cell; VEGF, vascular endothelial growth factor.

## 7 Cryoablation factors affecting immune effects

**Figure 2:**
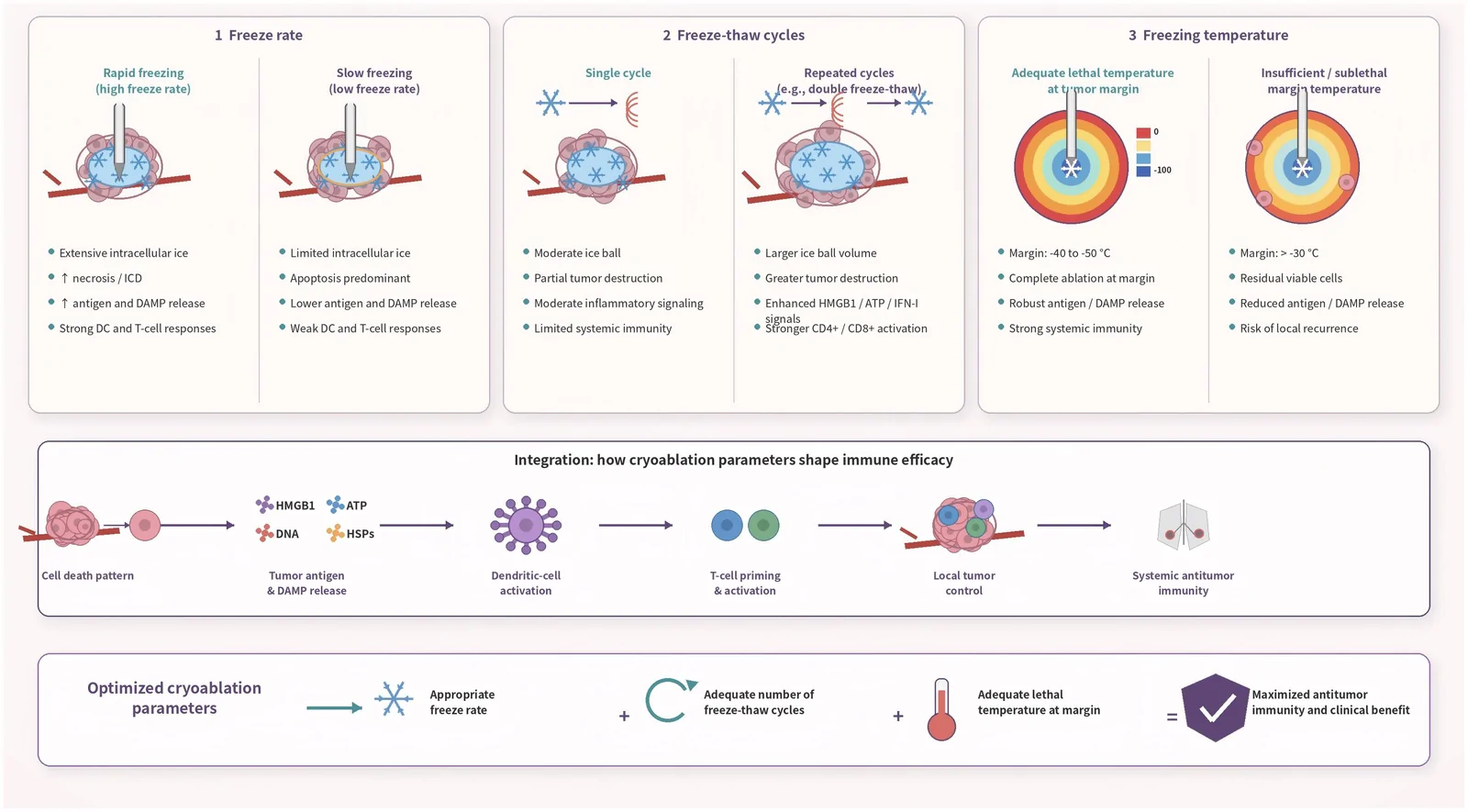
Key parameters affecting the immunological efficacy of cryoablation. Freezing rate, number of freeze-thaw cycles and lethal margin temperature jointly determine cell-death patterns, antigen/DAMP release and activation of the DC-T-cell axis, thereby influencing local control and abscopal immunity. (ICD, immunogenic cell death; DAMP, damage-associated molecular pattern; DC, dendritic cell; HMGB1, high mobility group box 1; ATP, adenosine triphosphate; IFN-I, interferon type I; CD4, cluster of differentiation 4; CD8, cluster of differentiation 8; DNA, deoxyribonucleic acid; HSPs, heat shock proteins).

The type of immune response triggered by tumor cryoablation is closely related to the pattern of cryo-induced cell death, and the selection of cryoablation parameters is a key determinant. For example, the marginal zone of a cryoablated tumor is often exposed to sublethal temperatures, under which cells usually undergo apoptosis. Therefore, systematic analysis of different cryoablation parameters is necessary to identify the key factors that elicit optimal immune effects and thereby improve immune outcomes (**Figure 2**).

### 7.1 Freezing rate

Freezing rate is a key parameter determining the efficacy of cryoablation, as rapid and slow freezing lead to distinctly different outcomes. Mechanistically, rapid freezing promotes intracellular ice formation and predominantly induces necrotic injury, whereas slower freezing allows greater cellular dehydration and may increase apoptosis at the marginal zone. Sabel and colleagues used high and low freezing rates to ablate mouse tumors and compared the results with surgical resection (91). They found that a high freezing rate induced tumor-specific immune responses, significantly reduced lung metastasis, and improved survival, while a low freezing rate increased regulatory T cells (Tregs), promoted lung metastasis, and reduced survival. These findings suggest that freezing rate affects the extent of necrosis, thereby altering the relative proportions of necrotic and apoptotic cells in the tumor microenvironment and modulating immune responses. Similarly, Yakkala and colleagues reported that in a mouse melanoma model, a higher freezing rate elicited stronger CD4^+^ and CD8^+^ T-cell responses and significantly prolonged survival after tumor rechallenge (26). In both breast cancer and melanoma models, rapid freezing consistently generated stronger tumor-specific immune responses, whereas slow freezing either increased Tregs or produced weak systemic protection (26, 91). However, Yakkala et al. did not observe cryoablation-induced apoptosis in any region on day 8 after ablation, indicating that further research is needed to elucidate the mechanisms of cryoablation-induced cell death *in vivo* and to determine the optimal protocol.

Although these animal studies clearly demonstrate biological sensitivity to freezing rate, their findings cannot be directly translated into a clinically optimal rate for each organ or tumor type. In clinical practice, cryoablation systems rarely deliver a uniform rate across an entire lesion, as the rate varies with distance from the probe, tissue perfusion, gas pressure, probe spacing, tumor composition, and adjacent structures; temperature measured at a single thermocouple cannot represent the complete three-dimensional thermal field. Furthermore, the rate that maximizes immunogenicity may not be the one that maximizes local tumor eradication. Therefore, clinical protocols must prioritize safe and complete tumor control while prospectively measuring immune endpoints, rather than deliberately leaving insufficiently ablated tissue to generate inflammation. Future studies should systematically collect data on temperature gradients, probe configuration, and tissue perfusion to establish correlations between freezing parameters and immune effects, thereby informing individualized treatment strategies.

### 7.2 Number of freeze–thaw cycles

The number of freeze–thaw cycles is an important parameter influencing cryoablation efficacy and the ensuing immune response. Studies have shown that repeated cycles significantly increase cell death rates (100), through mechanisms involving aggravated mechanical injury, enhanced vascular stasis, and expansion of the lethal zone. Clinical reports indicate that double-freeze cryoablation has positive effects in tumor treatment (101); compared with a single cycle, two cycles generate a larger ice ball and expand the area of cryoinjury, thereby enhancing tumor destruction (102).

In terms of immune effects, Takahashi and colleagues inoculated mice bilaterally with lung cancer or melanoma cells and performed one, two, or three cycles on one side, using the contralateral side to observe the abscopal effect (103). Contralateral tumor growth was slowest after two cycles, accompanied by significantly increased CD4^+^ and CD8^+^ T-cell proportions and proinflammatory cytokine secretion. In a bilateral Lewis lung cancer model, two cycles produced a stronger abscopal effect than one or three cycles, suggesting a non-linear relationship with an optimal window (103). These findings indicate that appropriately repeated freeze–thaw cycles help stimulate antitumor immune responses.

However, translating these animal findings into clinical practice faces substantial obstacles. Although two-cycle protocols are commonly used, organ-specific procedures vary considerably and are primarily designed for local control rather than immune optimization. Major challenges include inconsistent definitions of cycle duration, lack of standardization in active versus passive thawing, variable inter-cycle intervals, ambiguous partial versus complete coverage, and differences in single- versus multi-probe configurations. Reporting only the number of cycles is insufficient for cross-study comparisons. Future reports should include freeze/thaw durations, gas settings, probe number and geometry, image-defined ice-ball dimensions, estimated margin, thermometry data, and the proportion of viable tumor left untreated. Such standardized reporting will facilitate accurate assessment of the true impact of freeze–thaw parameters on immune outcomes and inform individualized clinical strategies.

### 7.3 Freezing temperature

Temperature parameters in cryoablation influence the efficiency of inducing antitumor immune responses by regulating cell death patterns and the intensity of inflammatory pathways. To ensure complete tumor destruction, clinical practice commonly uses lethal temperatures of -40 °C to -50 °C and ensures that the ice ball extends 1 cm beyond the tumor margin. However, this standard also limits the predictability and controllability of treatment (104).

Temperature exerts clear threshold effects on cell killing. Renal cancer cells are largely unaffected at -5 °C, show markedly reduced viability at -15 °C to -20 °C, and completely lose viability at -25 °C or below (105). Prostate cancer cells exposed to temperatures below -30 °C exhibit significant apoptosis within 30 minutes of thawing, peaking at 90 minutes and transitioning to necrosis after 6 hours, whereas at temperatures above -30 °C, apoptosis is delayed until 6–24 hours (106). Cryoablation also induces the secretion of proinflammatory cytokines such as TNF-α and IFN-γ, and the low-temperature environment (0–10 °C) synergizes with Kupffer cells to amplify inflammatory responses (107), suggesting that precise temperature control can induce more favorable antitumor immune effects through differential activation of cell death and inflammatory pathways.

However, clinical translation of these findings faces substantial obstacles. Cell survival depends on both temperature and exposure time, and the *in vitro* temperature threshold conclusions from renal and prostate cancer models cannot be directly extrapolated to complex clinical scenarios (105, 106). The temperature at the visible ice-ball edge is significantly higher than that in the central lethal zone, and constraints such as heat sink effects from adjacent large vessels and the need to protect vital organs often prevent uniform ablation margins; the nominal probe temperature therefore cannot represent the actual tissue temperature at the tumor boundary. In line with the standardized reporting framework proposed for freeze–thaw parameters, consistent documentation of core data—including device and probe specifications, lesion characteristics, thermometry and ice-ball imaging information, and immune sampling protocols—is essential for cross-study comparisons and for establishing immunity-guided, individualized cryoablation strategies.

## 8 Discussion

Cryoablation combined with immunotherapy provides a new synergistic strategy for tumor treatment. By releasing tumor antigens through local ablation and remodeling the immune microenvironment, it complements systemic immune intervention and shows substantial application potential. Based on a comprehensive review of recent literature, several key questions have emerged regarding the specific role of cryoablation in tumor treatment and require further study and discussion.

### 8.1 Biology-informed sequencing and patient selection

The optimal sequencing of cryoablation and immunotherapy remains unresolved, and no clinical consensus has been established despite substantial preclinical and clinical evidence supporting their synergistic antitumor effects. Three main timing strategies are currently used in clinical practice, each with distinct rationales, applicable scenarios, and levels of evidence.

The first and most common approach is ablation followed by immunotherapy, in which PD-1/PD-L1 inhibitors are initiated within 1–7 days after cryoablation. The rationale is to use the large amount of tumor antigens released by ablation as an endogenous *in situ* vaccine, and then to relieve immunosuppression through immune checkpoint inhibitors to efficiently activate effector T-cell clones targeting neo-antigens. This strategy is most suitable for patients with a safely ablatable lesion, limited imminent risk of systemic progression, an immune-cold or antigen-poor state, and the ability to start systemic therapy promptly after tissue injury. CASTLE-01 is an example of a planned ablation-first sequence, although the independent contribution of each treatment component remains uncertain (71).

The second approach, a newer trend, is immunotherapy followed by ablation, with CTLA-4 or PD-1 inhibitors given 3–7 days before the procedure. The rationale is to prime immune cells in advance so that they can immediately recognize and respond to antigens when they are released by ablation, thereby avoiding antigen clearance in an immunosuppressive microenvironment. This strategy is suitable for patients who have already benefited from ICI therapy but subsequently develop oligo-residual or oligoprogressive disease. In this setting, systemic therapy has already selected or expanded responsive T-cell clones, and local ablation can eliminate resistant reservoirs while providing new antigens to amplify systemic immune responses. BOOSTER provides the strongest clinical support for this disease-state-based sequencing strategy (55). For patients with primary ICI resistance, bulky disease, rapid progression, or a highly immunosuppressive tumor microenvironment, the addition of cryoablation alone is unlikely to rescue systemic therapy unless the resistant burden can be substantially reduced and suppressive pathways are also addressed.

The third approach is a cyclic or serial strategy. For patients with advanced multifocal metastases, one accessible lesion can be ablated while immunotherapy is maintained, and newly progressed oligo-lesions can then be ablated sequentially, thereby continuously maintaining systemic immune activation through staged antigen release (35). Serial antigen release could theoretically sustain clonal diversification and prevent antigen escape, but repeated procedures may also exhaust lymphocytes, destroy useful lymphoid tissue, delay optimal systemic treatment, or add cumulative complications. Evidence for this strategy is mainly observational (75), and each new lesion should be assessed for biopsy, molecular evolution, and the risk-benefit ratio of alternative local treatments rather than automatically ablated.

**Figure 3:**
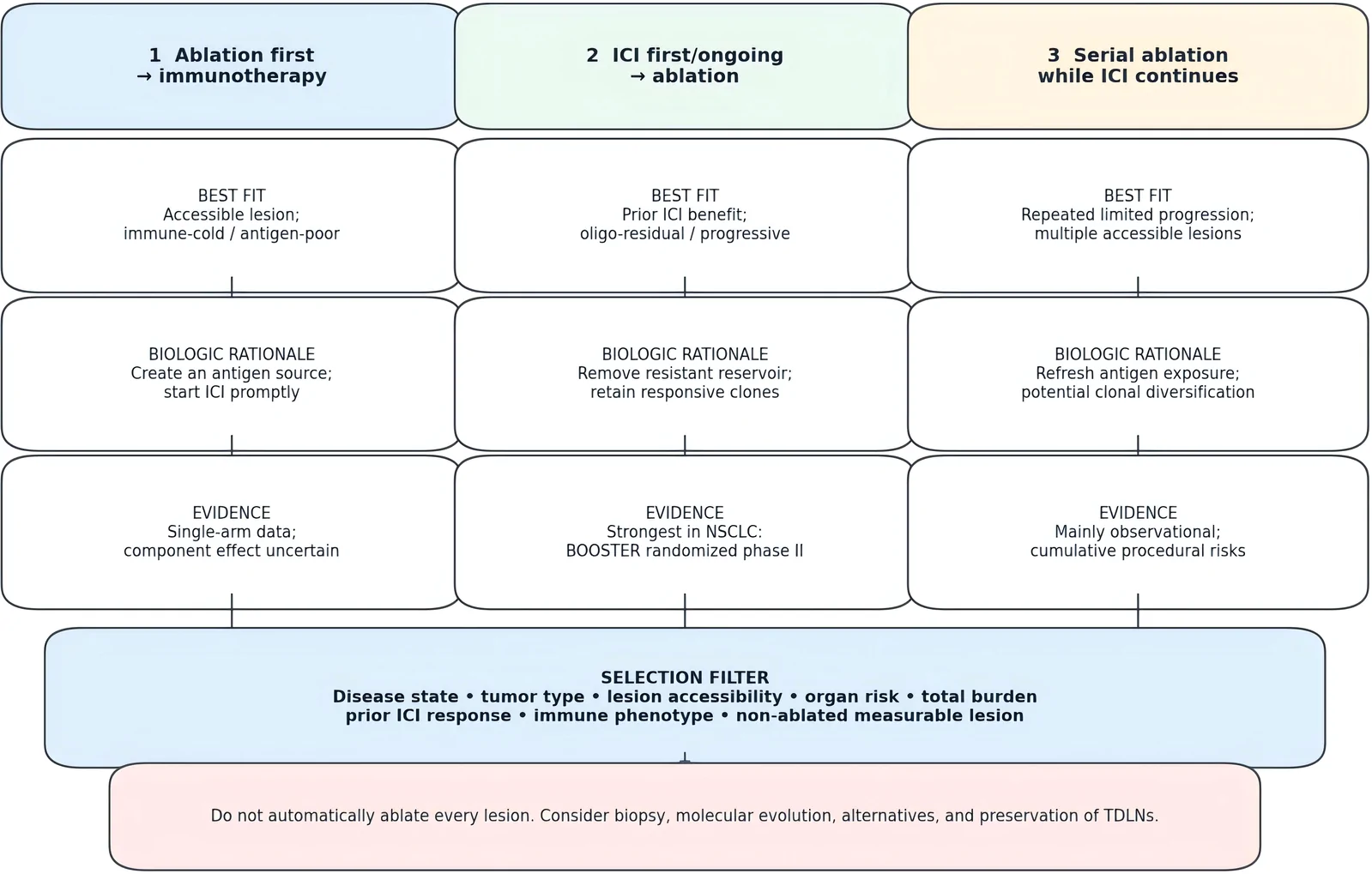
Sequencing strategies and patient selection framework for cryoablation combined with immunotherapy. Three main timing strategies are illustrated: ablation followed by immunotherapy (antigen source creation), immunotherapy ongoing followed by ablation (resistant reservoir removal), and serial ablation during continued immunotherapy (antigen refreshment). The framework integrates disease state, tumor type, lesion accessibility, organ risk, total tumor burden, prior ICI response, immune phenotype, and availability of non-ablated measurable lesions. Not all progressing lesions should be automatically ablated; biopsy, molecular evolution, alternatives, and preservation of tumor-draining lymph nodes (TDLNs) should be considered. (ICI, immune checkpoint inhibitor; NSCLC, non-small cell lung cancer; TDLNs, tumor-draining lymph nodes).

Tumor type modifies these choices, and the strength of recommendations should be calibrated to the evidence tier outlined above. NSCLC with oligo-residual disease has the clearest randomized support for ablation during ongoing immunotherapy, although cryoablation accounted for only a minority of the ablations performed in that trial (55). Liver-dominant melanoma and intrahepatic cholangiocarcinoma have encouraging but non-randomized data favoring ablation-first sequencing, and melanoma additionally provides the only lesion-selection rule with outcome data attached, namely preferential ablation of intrahepatic lesions no larger than 30 mm (71, 76). Prostate cancer supports the priming-first concept most directly at a mechanistic level, since CTLA-4 but not PD-1 blockade potentiated cryoablation in a hormone-sensitive model, suggesting that in immunologically quiescent tumors the choice of checkpoint may matter more than the choice of sequence (78); clinically, however, prostate data remain confined to pilot and phase I studies in which concurrent androgen deprivation or cellular therapy prevents attribution (79, 80). Renal and breast studies establish feasibility and reproducible immune modulation in the peri-ablation setting but do not define an optimal sequence, and renal cell carcinoma provides the explicit warning that immune activation and clinical benefit can dissociate (83–86). Pancreatic, colorectal, and bone or soft-tissue evidence is retrospective or preclinical, and no sequencing recommendation can be made; in these settings cryoablation should be combined with immunotherapy only within clinical trials (57, 95, 97, 98). Patient selection should therefore integrate disease state, lesion accessibility, organ risk, tumor burden, prior ICI response, immune phenotype, and the availability of non-ablated measurable lesions (**Figure 3**).

### 8.2 Predictive and pharmacodynamic biomarkers

No biomarker has been prospectively validated specifically for cryoablation-immunotherapy benefit. Candidate markers fall into four groups. Clinical and anatomical markers include oligoresidual or oligoprogressive state, the total metastatic burden, the number of involved organs, lesion size, LDH, performance status, and the availability of a safely ablatable lesion. In melanoma liver metastases, lower LDH, fewer metastatic organs, and ablated lesions no larger than 30 mm were associated with better outcomes, but these findings require validation (76).

Tumor and immune-tissue markers include PD-L1, baseline CD8/Treg ratio, dendritic-cell abundance, tertiary lymphoid structures, cGAS-STING competence, type I interferon signatures, CXCL10+ macrophages, CXCR3+ T cells, and T-cell receptor clonality (35, 36, 83, 87). Circulating candidates include ctDNA clearance, tumor-reactive T-cell clones, IFN-α/γ, HMGB1, HSP70, cytokine kinetics, and lymphocyte-to-myeloid ratios. Increased circulating PD-1/PD-L1 after cryoablation has been associated with poor prognosis in HBV-related hepatocellular carcinoma, illustrating that post-treatment biomarkers may identify adaptive suppression rather than benefit (74).

Procedural and imaging biomarkers may include three-dimensional lethal-margin coverage, residual perfusion, radiomics, metabolic imaging, and immune-PET tracers (96, 108, 109). The most informative designs will combine baseline and serial tissue, blood, and imaging with predefined sampling times, because a single post-ablation measurement can miss transient or delayed effects. Biomarkers should be tested as interaction markers that predict incremental benefit from adding cryoablation, not merely as general prognostic factors (109). **Table 4** summarizes candidate biomarkers across different domains, along with their proposed uses and current limitations.

**Table 4:** Candidate biomarkers for cryoablation-immunotherapy combinations.

Biomarker domain	Candidate markers	Proposed use	Current limitation
Clinical/anatomical	Oligoresidual/oligoprogressive state; burden; number of organs; lesion size; LDH; performance status	Identify patients with controllable resistant reservoirs and adequate systemic reserve	Mostly prognostic; cutoffs not validated across tumors (55, 76)
Tumor immune tissue	PD-L1; CD8/Treg ratio; DCs; TLS; cGAS-STING; type I IFN; CXCL10+ macrophages; CXCR3+ T cells	Predict antigen sensing, priming, and effector recruitment	Spatial heterogeneity and sampling-time dependence (35–37, 83)
T-cell repertoire	TCR clonality and expansion of tumor-reactive clones	Measure productive priming and systemic expansion	Requires paired tissue/blood and tumor-antigen linkage (87)
Circulating molecular	ctDNA clearance; HMGB1; HSP70; IFN-alpha/gamma; cytokine kinetics; soluble PD-1/PD-L1	Early pharmacodynamic response or adaptive suppression	No validated assay/time point; inflammatory confounding (74)
Imaging/procedural	Ice-ball margin; residual perfusion; radiomics; metabolic or immune- PET	Confirm lethal coverage and identify residual immunologically active tissue	Device and modality variability; immune imaging investigational (96, 108, 109)

DC, dendritic cell; HMGB1, high-mobility group box 1; HSP70, heat-shock protein 70; IFN, interferon; LDH, lactate dehydrogenase; PD-1, programmed death-1; PD-L1, programmed death-ligand 1; PET, positron emission tomography; TCR, T-cell receptor; TLS, tertiary lymphoid structures; Treg, regulatory T cell.

### 8.3 The role of tumor-draining lymph nodes

Third, the role of tumor-draining lymph nodes (TDLNs) remains an important question. In patients with lymph-node metastasis, cryoablation can directly inactivate lesions, relieve compressive symptoms, and reduce systemic tumor burden as a debulking method (18). Regarding immune-activating potential, existing case studies suggest that ablation can induce abscopal effects under specific conditions. After cryoablation of local lesions and involved lymph nodes, untreated distant metastases also shrink, suggesting that tumor antigens released by ablation can enhance systemic antitumor immune responses and provide a potential synergistic route for combination immunotherapy (90).

However, this treatment also carries clear risks. If a lymph node is not completely occupied by cancer cells, ablation may destroy the internal reticular scaffold and healthy immune-cell reserves of the lymph node, thereby directly damaging a core priming site for antitumor immunity (110). Recent studies have further confirmed that TDLNs are the key battlefield where PD-L1 inhibitors exert their effects. Premature or excessive ablation of TDLNs may deprive immune checkpoint inhibitors of the source of effector T-cell generation and markedly reduce systemic treatment response rates (110, 111). Therefore, it is necessary to define the population that can benefit from TDLN ablation. Indicators such as lymph-node metastatic burden, immune-cell infiltration ratio, and PD-Ll expression level may be combined to establish concrete criteria for when ablation is appropriate and when lymph-node structure should be preserved, thereby avoiding overtreatment. Future studies should also explore parameter standards for partial or gentler ablation strategies, inactivating nodal metastases while preserving immune drainage function and cellular reserves as much as possible, and balancing local control with systemic immune benefit.

### 8.4 Evidence limitations and future trial design directions

Current evidence on cryoablation combined with immunotherapy derives predominantly from animal models, single-center small-sample cohorts, heterogeneous combination regimens, and small-scale trials. Positive findings are susceptible to selection bias—for example, the inclusion of patients with low tumor burden, accessible lesions, and favorable immune phenotypes—whereas negative studies are equally informative, as they indicate that antigen release alone is insufficient to generate systemic benefit in tumors with poor immunogenicity or a highly suppressive microenvironment (9, 69, 83). Consequently, future trials should incorporate randomization within well-defined disease states, include systemic treatment controls, stratify by tumor type and prior response, and prespecify endpoints that distinguish locally ablated lesions from non-ablated measurable lesions, so as to overcome the current fragmentation and risk of bias in the evidence base.

### 8.5 Safety and treatment optimization

The safety assessment of combination therapy must address both procedure-related complications (such as pneumothorax, hemorrhage, pain, infection, organ-specific injury, cryoshock, and post-ablation inflammatory syndrome) and systemic immune-related toxicities (including immune-related hepatitis, pneumonitis, colitis, endocrinopathies, and dermatologic events) (112, 113). Attribution becomes particularly challenging when symptoms of the two categories overlap—for instance, postprocedural fever and elevated cytokines may stem from either infection or immune activation (112). Therefore, clinical trials should prespecify clear attribution algorithms and observation windows, and thoroughly document the severity, management measures, treatment delays, and recovery outcomes of all adverse events (113). Moreover, despite growing clinical experience with various ablation protocols, the substantial interindividual variability in post-ablation immune responses poses a challenge to defining an optimal strategy (92). Future research should focus on identifying the “optimal immunostimulatory dose,” which may differ from conventional ablative doses aimed solely at maximizing cell killing. Meanwhile, although combinations with immune checkpoint inhibitors (PD-1/PD-L1 and CTLA-4 inhibitors) have shown promising antitumor efficacy, the optimal drug combinations, timing, and dosing schedules remain non-standardized, with disparate recommendations across studies and no consensus reached to date (48). Rigorously designed randomized controlled trials are urgently needed to clarify these issues (114).

## 9 Conclusion and prospects

Cryoablation combined with immunotherapy provides a new synergistic strategy for tumors. Cryoablation induces immunogenic cell death (ICD), releases tumor antigens and DAMPs, remodels the tumor microenvironment, converts cold tumors into hot tumors, and thereby stimulates systemic antitumor immune responses. However, immune effects induced by cryoablation alone are limited in intensity and duration and are insufficient for comprehensive control of metastasis and recurrence. Immunotherapeutic approaches, including immune checkpoint inhibitors such as PD-1/PD-L1 inhibitors and CTLA-4 inhibitors, adoptive cell therapy, Toll-like receptor agonists, and tumor vaccines, can complement cryoablation mechanistically and enhance antitumor effects.

Although several recent reviews have summarized the general mechanisms and preliminary efficacy of this combination strategy, few have systematically translated mechanistic insights into clinically actionable frameworks. The present review distinguishes itself by moving beyond descriptive synthesis to provide four clinically oriented contributions: (1) a parameter–response framework linking cryoablation settings to immune outcomes; (2) a disease-state-based sequencing algorithm integrating prior ICI response and lesion burden; (3) a risk–benefit analysis for tumor-draining lymph node management; and (4) a tumor-by-tumor evidence appraisal graded by evidence tier, such that the strength of each recommendation is explicit and the tumor types for which no clinical data exist are clearly identified. By framing these underexplored bottlenecks as solvable clinical questions rather than mere knowledge gaps, we aim to bridge the gap between mechanistic understanding and individualized treatment planning. We also highlight remaining challenges and outline priority areas for future research, including biomarker-guided patient selection and prospective validation of sequencing strategies.

Existing preclinical and clinical studies have examined this combination across NSCLC, hepatobiliary cancer, melanoma, prostate cancer, renal cell carcinoma, breast cancer, pancreatic cancer, colorectal cancer, and bone or soft-tissue tumors, but the strength of that evidence is markedly uneven. Randomized data exist only in NSCLC, and that trial tested local ablation as a class rather than cryoablation specifically; prospective single-arm data with immune correlates exist in intrahepatic cholangiocarcinoma, melanoma, prostate, renal and breast cancer; pancreatic, colorectal, and bone or soft-tissue disease are supported only by retrospective series or preclinical models; and no clinical data at all are available in head and neck, gynecologic, esophageal or most remaining malignancies. Safety has nevertheless been consistent across tumor types, with procedure-related events remaining organ-specific and immune-related events remaining those expected of the checkpoint agent used, although the single grade 4 hepatic toxicity observed among five patients in a perioperative dual-blockade breast study is a reminder that the toxicity ceiling is set by the intensity of the immunotherapy backbone rather than by ablation. Nevertheless, the field still faces many challenges. Optimal cryoablation parameters, including freezing rate, number of freeze–thaw cycles, and freezing temperature, have not been unified. Sequential strategies for combination therapy, including ablation before immunotherapy, immunotherapy before ablation, and cyclic sequential approaches, lack standardized consensus. The boundary between preservation and ablation of TDLNs remains unclear. Tumor heterogeneity and individualized immune phenotypes also lead to significant differences in efficacy. Moreover, most current studies are still at the basic or early clinical stage, and large prospective randomized controlled trials are lacking.

When combined immunotherapy is used, reducing drug dosage or identifying novel innate immune agents may help decrease adverse effects and drug resistance while improving anticancer efficacy. Future studies should focus on four areas: defining optimal immune-activating parameters for cryoablation; establishing individualized timing -decision models based on biomarkers such as HMGB1, IFN-γ, and T-cell subset ratios; exploring strategies that balance TDLN preservation with local control; and conducting multicenter, large-sample, randomized controlled clinical studies to verify the generalizability and long-term survival benefit of combination regimens. These efforts will maximize synergy between cryoablation and immunotherapy and promote tumor treatment toward a precise, efficient, and low-toxicity era of combined immunotherapy.
